# RETRACTED ARTICLE: A conversational analysis of aging in China from a cross-section of the labour market: a corpus-based study

**DOI:** 10.1057/s41599-022-01387-y

**Published:** 2022-10-17

**Authors:** Yonghe Xiao, Jingxuan Li

**Affiliations:** 1 School of Chinese Language and Literature, Jiangxi Normal University, Nanchang, China; 2 School of Foreign Languages, Nanchang Institute of Technology, Nanchang, China; 3 School of Foreign Languages, Hunan University of Technology and Business, Changsha, China

**Keywords:** Economics, Economics

## Abstract

Modern society is undergoing rapid technological growth and urbanisation. Despite the positive changes, there are still vulnerable categories of the population that cannot adapt so quickly to the new realities. The ageing process in the developed countries of Europe, America and Southeast Asia raises the issue of further labour market development. In this regard, it is vital to understand the linguistic picture of a quickly ageing labour market, such as China. Drawing on Conversation Analysis, this study aims to investigate the impact of the ageing process on the labour market and identify age-related trends in communication, behaviour and attitude. The focus is on the socio-economic context. The most important finding is that in most contexts, the language behaviour of ageing labour market participants leaned on three underpinning factors: age, social status and exposure to emotional pressure. Older adults in the Chinese labour market value their informal status, accept social hierarchy, follow strict etiquette rules, tend to self-victimise, and complain about feeling vulnerable. The present findings can help social workers in China improve care for ageing adults and allow other researchers to investigate older people’s participation in the labour market.

## Introduction

Despite many global challenges of our time, human society continues to develop through the acceleration of technological progress. Technology affects people’s social lives by reshaping their lifestyles, worldviews, and ways of working. Under the influence of these trends, it is especially important to highlight demographic changes. The efforts to build a better future and achieve stability facilitate the growth of internal migration. Meantime, individuals who want a successful career tend to put family and children lower on their list of priorities. The consequence is the population decline, which strongly affects the sustainable socio-economic development in the country, including in rural areas (Li, 2015). The perception of well-being does not necessarily resonate with long-term planning; as a result, there is little understanding of what life will be like in old age. Given the trend of steady population decline in developed countries, it becomes apparent that hardly anybody is concerned about the challenges older adults face at the junction of technological eras.

One of the underpinning factors of socio-economic development is the global ageing of the population, primarily in most developed countries. According to the UN data cited by researchers (Feng et al., 2019), 80 out of 186 developed countries have a fertility rate below the replacement level (less than 2.1 children per woman). According to most experts, restoring the population growth rate in developed countries is an unattainable goal in the coming decades (Ambrosi-Randić et al., 2018; Feng et al., 2019). At the same time, 80% of the elderly population will live in low- and middle-income countries. The ageing rate of China is higher than that of developed countries. China lags behind Japan in population decline, though the difference is slight (Chen et al., 2019; Wang, 2019; Zhang, 2021). Life expectancy shows a rise in most countries, including China. It rose from 73.04 years in 2000 to 78.11 years in 2010. In 2000, the population aged 65 and above accounted for 7.10% of the total population. In 2010, it was 8.87% and continued growing afterwards, reaching 9.6% in 2015. The expectation is that the over-60 population will make up 27.6% of the total population by 2050. The old-age dependency ratio increased from 0.13 to 0.47 over the same period (Cheng et al., 2019).

The ageing process in China is uneven across regions and is accompanied by internal migration (Jiang, 2018; Chen et al., 2019). During the 1995/2000 period, the internal migration across provinces amounted to 32.28 million people. In 2005/2010, it was 54.99 million people, and this process retains a steady upward trend (Chen et al., 2019). Most researchers point out that internal migration in China is mainly directed from rural areas to cities and involves young and working-age populations (Chen and Hu, 2021; Ma, 2018a). The movement of the population between provinces is different. In 2000/2015, there was a shift from the central and western provinces with less developed industries to the eastern coastal ones, where the largest trade, administrative, and manufacturing centres are located. This process was due to more intensive ageing in the provinces-donors of the population (Henan, Sichuan, Anhui, Jiangxi, etc.). Prior to this, ageing was more rapid in the eastern coastal regions; researchers associated it with the increased life span in economically and socially developed provinces (Harmel and Yeh, 2015; Li et al., 2018; He et al., 2019).

The problem is that the ageing population in China is not wealthy, and the level of social security is much lower than in developed countries. Its per capita GDP in 2001 was estimated at less than 3 thousand dollars; for comparison, it was 10–20 thousand dollars in South Korea at the time (Wang, 2019). The government had a plan to expand the pension system and cover the rural population for the period until 2020. Researchers doubt the effectiveness of this pension system, considering the rapid pace of ageing and labour market shrinkage (Bai and Lei, 2020; Zhang, 2021). The concern is that China may fall into a middle-income trap (Feng et al., 2019; Zhou et al., 2020).

Among the current problems of China and other counties is the digitalisation policy, namely the level of computer literacy education among the older generation (Schmidt-Hertha and Strobel-Dümer, 2014). Digitalisation has diverse and highly interconnected effects on employment. It causes polarisation of the labour market across many countries. On the one hand, digitisation boosted demand for high-skilled tech-savvy labour. On the other hand, it entailed a stark decrease in demand for low- and medium-skilled workers, including older adults, mostly without higher education degrees (Bejaković and Mrnjavac, 2020). This problem is typical of resource-based economies, including China. Without proper oversight, difficulties in intergenerational communication risk turning into tensions that can destabilise social relations.

One way to solve the above problems and systemise the information is Conversation Analysis. This method allows investigating social relations and behavioural patterns derived from ethnomethodology through everyday communication using linguistic constructions (Pain, 2020). In this study, Conversation Analysis was applied to examine the role of the rapid ageing process in the changing labour market. Technological advancement changes the labour market, imposing new requirements on educational backgrounds and redefining the linguistic experience of social subjects (Liu et al., 2021).

## Literature review

The ageing process refers to age-related alterations. The affected areas include, among other things, cognitive functions, decision-making, social interaction, and personal needs (Ambrosi-Randić et al., 2018; Harmel et al., 2019). The researchers point out that, despite changes in concentration and focus, most characteristics associated with cognitive ability (i.e., impulsivity and attention span) are similar between middle-aged and old-age individuals (Lufi et al., 2015).

Previous research shows a link between a senior person’s emotional intelligence and well-being and highlights the importance of education (Shinan-Altman and Werner, 2019). A good educational background and a deeper engagement with others maintain the quality of life and affect employment in old age (Ambrosi-Randić et al., 2018; Chen et al., 2016). In the Chinese context, this is a controversial issue, for most ageing residents may not have a college degree and may have a relatively low level of emotional intelligence (Ko and Yeung, 2019a). This problem requires extensive research with large geographic areas and representative samples in focus.

Economic reforms in China caused the female labour supply and education levels to increase. However, the country is still lagging behind the OSCE member states and developed countries in Southeast Asia (Chen et al., 2019; Ma, 2018b; Qian, 2021; Wang, 2019). An increase in the number of older people can considerably affect political choices and voting results, which depend on older adults’ economic satisfaction and social ties (Harmel and Yeh, 2015). Social participation and engagement in paid work are the most crucial factors in maintaining social resilience. In many developing countries, ageing populations can heavily influence decision-making and choice processes (Lukyanets et al., 2021; Webster and Pierce, 2019). Ageing contributes to the formation of multi-age groups where relationships are age-driven. Researchers point out that age-related prejudice in the workplace stems from cultural stereotypes and intragroup favouritism (McNamara et al., 2016). In China, authorities created a particular niche in the labour market for older people (He et al., 2019; Li et al., 2021).

The most common sources of inequality are gender, age, and educational background. According to several studies, gender inequality received the most attention, and age-related issues came to the forefront in many countries (Fraile and Fortin‐Rittberger, 2020). There can also be ethnic, language-based and religious inequalities causing various conflicts, including in the labour domain. In China, however, this issue is not a serious concern because the ethnic and linguistic differences represent the building blocks of Chinese mentality, which has various ethnicities at its core. The most noticeable differences prevail in the western regions, such as Tibet and Xinjiang. Owing to their physical inaccessibility and low population density, these regions are often ignored, nevertheless (Li, 2021).

As communication between age groups in China is guided by Chinese traditionalism, it is crucial to investigate age-related changes in the country’s demographic profile. The most suitable research method in this regard seems to be Conversation Analysis. It allows one to examine patterns of language behaviour based on inductive conclusions (Pain, 2020).

Despite the wide use of Conversation Analysis in psychotherapy and pragmatic linguistics research, it is almost impossible to find studies that would capture the problem of ageing and age-related social changes (Liu et al., 2021; Yoon and Stine-Morrow, 2019; Yu and Liang, 2018). The present study seeks to narrow this gap.

This study aims to identify age-related trends in communication, behaviour, and attitude by looking into the Chinese labour market. The novelty of the study is the application of Conversation Analysis to a large corpus of utterances, which cover different contexts and relationships associated with the ageing workforce issue.

## Methods

The study employs several methods to examine the main trends in and socio-economic contexts of communication, behaviour and attitude throughout the ageing process in China. Patterns and hypotheses to-be-tested in corpus data analysis were determined by Conversation Analysis (CA), an inductive qualitative research method (Liddicoat, 2021; Pain, 2020).

Given the size of the country and regional variations in social, economic and demographic conditions, the corpus data analysis procedure involved applying a filter to recorded conversations (Liddicoat, 2021). The focus was on records made in the cities (centres of internal migration attraction) where urban residents and rural migrants co-exist. The analysis of statements made by older residents and reactions from their interlocutors made it possible to comprehensively examine the social and linguistic patterns of communication among older people in the labour market. Records in the study corpus covered the following contexts: recruitment, production decision-making, work-related conflict resolution, financial issues, orders received, communication with family members or friends in the workplace and after work, job prospects, employment and related concerns/expectations.

The selection criteria for the study population were Chinese residents aged 60 and older (i.e., the elderly). The rationale behind this choice stems from the statutory retirement age. In China, the official retirement age is 60 years for men, 50 years for women, and 55 years for white-collar workers (OECD, 2021). According to the latest UN Population Division data, the life expectancy in China is as follows: Male, 75.3 years; female, 80.15 years; total life expectancy, 77.47 years (Worldometer, 2022). No regard was given to ethnicity, social environment, and conflicts on ethnic or religious grounds (they are not typical of Chinese society). The Chinese language has many dialects. To avoid misunderstanding among people who can only speak their native dialects, all participants in the study spoke Mandarin, the official spoken language of China taught at schools as a language of interregional communication.

The second part of the study involved employees, employers, family members or friends of the elderly Chinese with whom they discussed labour relations or financial/social issues. Recording conversations with these categories of people at home would be problematic. Therefore, the recording took place on the streets, at work, and in previously agreed places. The researcher arrived at or was present in specific locations at all times to record conversations between participants in a relaxed and unconstrained environment. Regarding survey credibility, one cannot guarantee the accuracy of the information given by the respondents.

The corpus of spoken data underwent analysis to determine repetitions of utterances that reflect trends in behaviour, context and social status of the interlocutors. The said patterns were then exposed to quantitative and correlation analyses to determine if there was a relationship with the context.

In this study, methods for questioning and collecting statistical data were used. The study population included 2271 people aged 60 to 65 and 1564 people older than 65. The total number of participants was 3835. The sample has a relatively equal number of locals and migrants per city, as well as an approximately equal number of men (*n* = 1902) and women (*n* = 1933). More information is presented in Table 1.

**Table 1:** (a) Sample demographics (part 1); (b) Sample demographics (part 2).

(a)	Nanjing		Suzhou		Chengdu	
	Men	Women	Men	Women	Men	Women
60–65	91/89	101/81	92/88	89/87	101/81	88/86
65 more	45/44	61/51	51/48	50/47	61/54	63/60

(b)	Zhengzhou		Shanghai		Beijing	
	Men	Women	Men	Women	Men	Women
60–65	99/91	102/71	101/100	111/101	115/106	99/90
65 more	59/59	62/57	89/76	101/85	88/79	90/81

Each core participant (Chinese citizen) had 7 to 16 interlocutors; recorded conversations included 2 to 5 people. There were 18 to 39 separate conversations per core participant. The context of a conversation was considered an independent variable.

Generally, the study was conducted by a team of authors from the Jiangxi Normal University. All recordings were taken between May and July 2021 in 6 cities across the central and western provinces of China: Nanjing and Suzhou (Jiangxi province), Chengdu (Sichuan province), Zhengzhou (Henan province), Shanghai, Beijing (centrally subordinated cities). The process involved enthusiasts and student assistants specially prepared for the Conversation Analysis who joined the study.

The timing of the study allowed minimising the chance of exposure to COVID-19. There were no pandemic restrictions imposed on production, trade and transportation sectors at the time, which could affect the research results.

All recordings were saved in a database, along with demographic profiles of the core participants. The database was transcribed and examined for repetitive statements, behaviours, contexts, or any combination thereof. The findings were verified using descriptive statistics and correlation analysis.

Data analysis was based on an inductive approach. The frequent patterns identified through the study of individual data sets underwent a statistical evaluation to establish whether they were statistically significant. This process involved two stages.

The first stage was *data encoding*. The correspondence between questionnaire questions and specific variables was established with the conversion key, which also helped to convert values to code numbers. The encoding process was done in Microsoft Excel 2019. Descriptive statistics were used to determine the frequency and density of the patterns.

The second stage was *Pearson’s correlation analysis*. According to the chi-square test results, the data sets are normally distributed and contain an equal number of variables.

Based on the results of sequential verification, less frequent patterns and those with statistically insignificant correlation coefficients were excluded from further analysis. The threshold frequency is 70%, and a correlation coefficient (r) between variables has to be at least 0.7. Statistical data processing was conducted through IBM SPSS 26.0. Data presentation was performed using Microsoft Excel 2019.

The study was conducted in accordance with the World Medical Association Declaration of Helsinki. The research procedure was approved by the Ethics Committees of Jiangxi Normal University. All participants in the study, including interlocutors, gave their consent to participate and were guaranteed confidentiality. Given the spontaneous nature of communication, the researchers maintained a constant hidden presence in research locations to record conversations whenever they took place without letting the participants know. Some participants, in particular production managers and other employees with delegated power, asked permission to view the content of the recordings for sensitive personal data. Such access was provided in all cases. All individuals approved the complete versions of the records.

No personal information (except demographic data) was collected, saved or used. There is no means of identifying participants involved in the conversation. The study thus guarantees the preservation of personal secrecy.

## Results

Based on the results obtained across various communication contexts, there are three factors that underpin the trends in language behaviour among older people (Table 2). These factors are age, social status, and stress exposure.

1. *Age ranking*. Some patterns were age-driven and varied depending on the age of interlocutors. These include greetings, rhetoric before the rest breaks, the presence/absence of speech repairs, the nature of lexemes and expressions used for clarification purposes, and time wait for the interlocutor to finish speaking.
2. *Ranking by social status*. The participant behaviours varied by the social status of the interlocutors. Trends include the refusal to criticise interlocutors with a higher social status and being silent when they raise their voices or make contradictory statements or accusations. These patterns of behaviour correspond to the interaction between people where one actor is younger than the other (Veresha, 2016). In this case, the core participants act as someone younger in this equation and interlocutors with a higher social status appear to take the role of an older actor.
3. *Exposure to emotional pressure*. There was also a conflict-driven variation in patterns. Trends include the reduced length of the utterance, the absence of speech repairs, a lowering of the voice, and a refusal to criticise. This behaviour was exclusively toward socially higher-ranking interlocutors. There were no statistically significant patterns in behaviour towards younger and age-similar interlocutors with similar or lower social status.

**Table 2:** The frequencies of behavioural patterns (the percentage of utterances in the corpus) and their correlation with the context of the conversation.

Patterns	Frequency	Correlation with context
Age ranking		
Traditional-style greetings	89.17%	0.756
Statements before rest breaks	87.14%	0.802
Pronounced speech repairs	91.02%	0.91
Time wait for the interlocutor to finish speaking	92.71%	0.761
Ranking by social status		
Refusal to criticise the socially superior interlocutor	95.43%	0.71
Being silent when a socially higher interlocutor raises their voice	80.54%	0.769
Younger person-older person communication style	91.66%	0.813
Exposure to emotional pressure		
Reduction in utterance length	97.09%	0.795
The absence of speech repairs	79.22%	0.778
Lowering of the voice	77.29%	0.86
Refusal to criticise	91.20%	0.892

The frequent behavioural and linguistic patterns reflect the current beliefs or values of the older population in the Chinese labour market. These beliefs and values are depicted in Table 3.

1. It seems that informal status is important for the elderly, as evidenced by a strong correlation between linguistic/behaviour patterns listed below and the context in which they manifest themselves. When interlocutors recognise their informal status (i.e., behave in a manner that is considered ‘traditionally polite’, wait a long time before starting to speak, and use polite forms of speech repairs), the elderly exhibit some vivid and consistent patterns of behaviour. For instance, they tend to express satisfaction, praise the interlocutor, and offer life advice. These forms of response are traditionally accepted and expected in this context.
2. Older people in the Chinese labour market tend to follow strict etiquette rules in personal communication. To be more specific, they employ the traditional forms of politeness in virtually all communication contexts where production, employment or financial issues are involved. Informal constructions emerge only with younger interlocutors whose social status is equal to or higher than that of the core participant. When talking to older people and those with higher social statuses, the elderly participants exhibited a tendency to generate shorter utterances.
3. Older Chinese people tend to self-victimise and feel insecure. The dominant linguistic/behavioural patterns include not responding to criticism, speaking in shorter sentences, silently receiving instructions/orders, avoiding discussions that touch upon senior officials, and not speaking of injustice with someone other than peers and those having similar social status. The likely reason behind such behaviour is the fear of punishment, social maladjustment, and job loss.

**Table 3:** The frequencies of beliefs/values (the percentage of utterances in the corpus) and their correlation with the context of the conversation.

Patterns	Frequency	Correlation with context
Preserve the informal status		
Express satisfaction	84.74%	0.912
Praise the interlocutor	88.47%	0.973
Offer life advice	75.90%	0.876
Adhere to hierarchical communication rules		
Use traditional forms of politeness	98.70%	0.762
Use informal constructions with younger people	88.75%	0.783
Produce shorter utterances	81.05%	0.788
Self-victimise and feel vulnerable		
Do not respond to criticism	98.05%	0.789
Speak in shorter sentences	91.87%	0.71
Receive instructions silently	86.44%	0.709
Avoid discussing the behaviour or words of a senior	97.51%	0.897
Do not speak of injustice with someone other than peers and those having similar social status	96.42%	0.78

## Discussion

The problems of socialisation and social adaptation of elderly employees are understudied because most developed countries are more concerned with pensions and medical support expenses (Fraile and Fortin‐Rittberger, 2020; Harmel et al., 2019). They appear to be more urgent in China, given the following circumstances. China failed to reach a sufficient level of national wealth per inhabitant, and coverage of the country’s pension system is uneven (Li et al., 2018; Lukyanets et al., 2021; Wang, 2019). Therefore, the socialisation of older people is crucial for preserving the workforce and maintaining a healthy psychological climate in a country with a rapidly ageing population (He et al., 2019; Li et al., 2021).

Some studies indicate that older people strive to remain in the younger age group for as long as possible. They perceive the transition to older adulthood as happening at later ages. Even 65-year-olds believe that a person becomes old at age 70 and higher. The desire to ‘push’ older transitions into the future and feel younger than their current age is a common psychological sign of age-related changes (Shinan-Altman and Werner, 2019). In an environment where ageing is associated with the loss of social opportunities and social uncertainty, one can expect an even stronger tendency among older adults not to admit their actual age and related changes.

The gradual changes to social ties in a traditional Chinese society require more thorough research. Traditionally, respect for and support of older family members are the most important values (Li et al., 2021). The increasing number of older adults, however, raises concerns about whether their relatives will have the ability to take care of them, especially in families with multiple children (Chen and Hu, 2021; Liu, 2021; Zhang, 2019). These changes can gradually make older adults more socially isolated, but if they maintain jobs, they could be more independent (Caliendo et al., 2019; Ma, 2018a, 2018b). The present study shows how important it is to preserve the informal status of an older person in the workforce. Similar results were presented in other ageing studies from China (Dong et al., 2018; Qian, 2021).

Another critical but poorly studied factor influencing the labour market is the internal migration of older adults. Some scholars emphasise the importance of this phenomenon for understanding the ageing labour market, especially in China with its continuing urbanisation, but the research on this topic is scarce (Bai and Lei, 2020; Feng et al., 2019; Harmel et al., 2019). Rural-to-urban migration leaders are young and middle-aged people seeking opportunities to make more money. The recruitment dynamics for people of different ages and the vertical dynamics of the labour force (older people occupy higher positions) also remain underexplored (Lu and Liu, 2019; McNamara et al., 2016; Wang, 2019).

Education and technological advances are core to understanding the vertical ageing dynamics in the labour market. The introduction of increasingly complex skill-intensive technologies into production and the widespread use of Artificial Intelligence will likely cause qualitative changes to the labour market in the next 30 years (Ko and Yeung, 2019a; Zhou et al., 2020). Women and older people with poor education and low income will be hardest hit by job cuts. Researchers expect China to replace 278 million workers (35.8% of all currently employed) with AI by 2049 (Zhou et al., 2020). The increasing older population may find itself unemployed, while social security and even social peace issues may become very acute. Some Western countries consider the possibility of installing a guaranteed basic income as a solution (Fraile and Fortin‐Rittberger, 2020; Webster and Pierce, 2019). China, however, cannot afford this strategy due to a tenfold lower gross national product per capita (Zhang, 2021).

As seen from the present findings, modern China has not yet recognised the above risks and the attitudes toward old employees remain unchanged, both at work and in the family. A tendency toward victimisation, self-perception of vulnerability, and acceptance of hierarchy and one’s place within it suggest the gradual awareness of the impending crisis by older Chinese. The relationship between hierarchical status and age at work in China is traditionally strong (Ko and Yeung, 2019b; Voss et al., 2018). A person can occupy higher posts only upon reaching a certain age, but this restriction is related more to traditions rather than logic. Things are currently changing in this regard, such that younger individuals have chances to occupy a socially significant post with the right to make decisions (Cheng et al., 2019). Older workers may perceive these changes as a threat to their previous status and believe that a younger boss will not tolerate having them in the workplace (Zhang, 2019). From this perspective, the adherence to conservative means of linguistically expressing loyalty and finding one’s place in the hierarchy may be a form of self-defence older adults use to handle the changing reality. The adoption of digitalisation programs for the elderly may prevent senior employees from retiring. Examples are computer literacy and advanced technology courses. Such initiatives are usually put forward at the local level. The government contributes by creating easy-to-use digital platforms for older people, but the large-scale effect is yet to come (Guo et al., 2022). Another problem with digital education programs for older employees is that Chinese readers experience age-related reading difficulty without age differences in the word-frequency effect (Wang et al., 2018). Older individuals enroled in digital education courses feel insecure when not motivated (Yap et al., 2022) and may experience stress when using technology. It is best not to worsen the situation and motivate older employees to learn digital technologies, thereby raising their social confidence and self-esteem (Martínez-Alcalá et al., 2018; Pan and Jordan-Marsh, 2010).

This study suggests that the traditional Chinese system of values and its focus on age and respect for the elderly is still in place. Therefore, the centuries-old approach to older people as custodians of experience and knowledge may still influence the labour market and slow down the psychological and social transformations in the coming decades (Ko and Yeung, 2018; Zou et al., 2018).

The study has several limitations. First, the Conversation Analysis cannot be considered a full-fledged quantitative study, even coupled with a quantitative analysis of linguistic and behavioural patterns. Second, the present study depicts a specific linguistic and social reality perceived and formed by people through communication, not statistically verified factors from a representative sample. Another limitation is the small number of cities. Future research will focus on other geographic areas, such as rural regions with population decline. Particular emphasis should be on autonomous regions with ethnic, linguistic, and religious differences defining the local social policy and public sentiment.

## Conclusion

The study aimed to narrow the gap in academic research on ageing in the labour market by using Conversation Analysis on a sample of senior employees in China. The goal was to identify age-related trends in communication, behaviour and attitude in China. The corpus of spoken utterances was collected from 3800 individuals in the central provinces of China and analysed using an inductive method. The patterns of behaviour were examined using descriptive statistics and correlation analysis. The results show three factors underpinning the contextual language behaviour of older Chinese in the labour market: age, social status, and exposure to emotional pressure. Older working individuals in China strive to maintain their informal statuses, follow strict etiquette rules, and demonstrate tendencies to self-victimise and feel insecure. The results of the study can be used to plan effective measures to improve public well-being among at-risk old-age groups and as a platform for further research.

## Data Availability

Data will be available on request.
